# Effect of Winning Experience on Aggression Involving Dangerous Fighting Behavior in *Anastatus disparis* (Hymenoptera: Eupelmidae)

**DOI:** 10.1093/jisesa/ieaa038

**Published:** 2020-05-27

**Authors:** Peng-Cheng Liu, De-Jun Hao, Hao-Yuan Hu, Jian-Rong Wei, Fan Wu, Jie Shen, Shen-jia Xu, Qi-Yue Xie

**Affiliations:** 1 The College of Life Science, Anhui Normal University, Anhui Province, China; 2 The College of Forestry, Nanjing Forestry University, Jiangsu Province, China; 3 The College of Life Science, Hebei University, Hebei Province, China

**Keywords:** aggressive behavior, dangerous fighting pattern, parasitoid wasp, transcriptomic analyses, energy

## Abstract

Aggressive behavior is widely observed in animal species for acquiring important resources and usually includes both dangerous and nondangerous fighting patterns. Only a few species show dangerous fighting patterns that are defined by fights ending with contestants being severely injured or killed. Prior experience, an important factor in many species, has been demonstrated to affect a contestant’s subsequent fighting behavior. Few studies have focused on the effect of experience on aggression involving dangerous fighting patterns. Here, an egg parasitoid wasp, *Anastatus disparis,* which shows extreme and dangerous fighting behavior to acquire mating opportunities, was used as an experimental model. Our results showed that the fighting intensity of the winning males significantly decreased subsequent fighting behavior, which was inconsistent with general predictions. Transcriptomic analyses showed that many genes related to energy metabolism were downregulated in winners, and winners increased their fighting intensity after dietary supplementation. Our study suggested that fighting in *A. disparis* is a tremendous drain on energy. Thus, although males won at combat, significant reductions in available energy constrained the intensity of subsequent fights and influenced strategic decisions. In addition, winners might improve their fighting skills and abilities from previous contests, and their fighting intensity after dietary supplementation was significantly higher than that of males without any fighting experience. Generally, in *A. disparis*, although winners increased their fighting ability with previous experience, the available energy in winners was likely to be a crucial factor affecting the intensity and strategic decisions in subsequent fights.

Most animal species show aggressive behavior to acquire important resources, such as food, breeding sites, shelter, and mates (Hardy et al. 2013). According to the literature, aggressive behavior is usually classified as nondangerous and dangerous fighting patterns (Enquist and Leimar 1990). The pattern of dangerous fighting is defined by fights ending with contestants being severely injured or killed. Because injury is energetically costly (Lane and Briffa 2017), individuals of most species usually tend to avoid conflict escalation, giving up before being injured and thus following a nondangerous fighting pattern (Maynard Smith and Price 1973, Maynard Smith 1982, Mesterton-Gibbons et al. 2017). Some species, such as *Epipemphigus niisimae* (Aoki and Makino 1982), *Frontinella pyramitela* (Austad 1983), and *Melittobia australica* (Abe et al. 2003, Innocent et al. 2011), have been reported to engage in dangerous fighting. Generally, predictions from the ‘hawk-dove’ game suggest that dangerous fighting is an evolutionarily stable strategy (ESS) only when the benefits of winning far outweigh the potential costs of conflict (Maynard Smith and Parker 1976; Enquist and Leimar 1987, 1990).

Prior fighting experience (Whitehouse 1997, Hsu and Wolf 1999, Jennings et al. 2004, Reaney et al. 2011, Schwartzer et al. 2013) is one factor, along with the fighting ability of each contestant (Parker 1974), value of the resource (Enquist and Leimar 1987, 1990; Mohamad et al. 2013; Stockermans and Hardy 2013; Mathiron et al. 2018; Liu and Hao 2019), density of competitors (Murray 1987, 1989; Innocent et al. 2011; Liu et al. 2017a), and relatedness of the combatants (Hamilton 1979; Reinhold 2003), that influences fighting decisions and consequences and is widely studied in many species, especially with nondangerous fighting patterns. Usually, prior experience affects a contestant’s fighting behavior by altering its estimated fighting ability or assessment of the resource value or the costs; it then affects fighting behavior during the match and the outcomes of later contests (reviewed by Hsu et al. 2006). Empirical reports proposed that prior experience losing fights decreased and winning fights increased the willingness and frequency of aggressive acts (Whitehouse 1997, Khazraie and Campan 1999, Hsu and Wolf 2001, Hsu et al. 2006). In addition, an increasing number of studies have shown that an individual’s previous winning and losing experiences induce physiological changes that modify behavior to influence the outcome of its current contest (Hannes et al. 1984, Booth et al. 1989, Elofsson et al. 2000, Øverli et al. 2004, Hsu et al. 2006, Schwartzer et al. 2013, Garcia et al. 2014). However, as fewer species show dangerous fighting patterns, most studies are related to experience affected by nondangerous fighting.

To acquire mating opportunities, an egg parasitoid wasp, *Anastatus disparis*, frequently engages in male-male combat following a dangerous fighting pattern near its emergence site (Liu et al. 2017a, Liu and Hao 2019). Here, *A. disparis* was used as an experimental model to study the effect of experience on aggression with dangerous fighting patterns. Prior fighting experiences usually could be divided into winning and losing experiences, and usually, prior winning experiences increase and losing experience decreased, the frequency of aggressive acts (Whitehouse 1997, Khazraie and Campan 1999, Hsu and Wolf 2001, Hsu et al. 2006). As the dangerous fighting pattern in this species leads to a loser sustaining injuries, injured males are considered to be losers with losing experience after fighting; otherwise, healthy males are considered to have a winning experience. As losers are injured, resulting in individuals who have difficulty moving and initiating attacks, our studies mainly focus on the effect of winning experience on aggression (Liu and Hao 2019). In addition, changes at the molecular level induced by experience might modify fighting behavior (Hannes et al. 1984, Booth et al. 1989, Elofsson et al. 2000, Øverli et al. 2004, Hsu et al. 2006, Schwartzer et al. 2013, Garcia et al. 2014), which was explored in the present study using Illumina-based transcriptomic analysis to further enrich knowledge and provide comprehensive insight.

## Materials and Methods

### Insect Cultures

A colony of *A. disparis* was established from a population that emerged from egg masses of *Lymantria dispar* collected in the wild. Eggs of *Antheraea pernyi* were provided as a substitute host for rearing *A. disparis* indoors (Yan et al. 1989, Li and Lou 1992, Liu et al. 2015). To prevent any mating and fighting experiences of wasps before the start of the experiment (Liu et al. 2017a, Liu and Hao 2019), parasitized *A. pernyi* eggs were isolated individually in polyethylene tubes (height: 7.5 cm; diameter: 1 cm) before wasp eclosion.

### Effect of Winning Experience on Aggression

To acquire winning experience, two 1-d-old newly eclosed males (from 9:00 a.m. to 10:00 a.m.) and a mated female were put into an arena at 10:00 a.m. We exposed only two males to one another when manipulating fighting experience which was logistically easier and controlled fight intensity. After 1 h (i.e., at 11:00 a.m.), the two males were isolated individually in polyethylene tubes (height: 7.5 cm; diameter: 1 cm). Fighting in *A. disparis* males typically ends with losers being injured when their feet or antennae are cut by the opponent’s mouthparts. The injury conditions of a contestant might be slight (e.g., the loss of part of an antenna or tarsus) or severe (e.g., the loss of most legs) (Liu and Hao 2019). In this experiment, the injury conditions of the two males in each group were checked with a microscope. Healthy males in the groups in which another male was injured (loss of more than one tarsus) were selected as acquiring winning experience for subsequent aggression assays. To acquire males without any fighting experience as a control treatment, males were isolated individually in polyethylene tubes (height: 7.5 cm; diameter: 1 cm) before the start of the aggression assays. Most adults eclosed during the peak eclosion period (from 9:00 a.m. to 12:00 p.m.), and male-male chasing and fighting near the emergence site were frequently observed. The least number of males reared on field-collected egg masses of L. dispar was four (Liu et al. 2017b), and fighting in *A. disparis* males belong to dangerous fighting pattern (Liu and Hao 2019). Thus, fighting intensity among four males might be appropriate measure for fighting behavior in *A. disparis.* Aggression assays lasted 3 h starting at 11:00 a.m., and four males (i.e., all winners or all without any fighting experience) were introduced into a cylindrical arena (height: 1 cm; diameter: 3.5 cm) containing a 1-d-old virgin female to estimate fighting intensity (Liu and Hao 2019). Previous study shows that female number in an arena have no significant effect on fighting intensity (Liu et al. 2017a). Thus, each arena contained one female. After 3 h, all males were removed and isolated individually in polyethylene tubes (height: 7.5 cm; diameter: 1 cm). The number of dead and injured males was recorded using a microscope; we also scored each visible male injury with a microscope according to a set of criteria (e.g., the loss of an antenna scored 0.5 points; detailed in Table 1) adapted from Murray (Murray and Gerrard 1984, 1985; Murray 1987, 1989). A male assigned a score of more than 7 was considered as severely injured. We then calculated the mean score of injuries per wasp and the proportions of injured males and severely injured males as fighting intensity in each arena. Respectively, 19 and 18 replicates for each treatment were performed. Besides, all tested adults did not get any dietary supplementation before beginning or during this experiment.

**Table 1.** Criteria used to score injuries to male *Anastatus disparis*

Type of injury	Score
Loss of part of whole antenna	0.5
Loss of part or whole tarsus	1.0
Loss of part or whole tibia	2.0
Loss of part of whole femur	3.0
Loss of part or whole coxa	4.0

### Transcriptomic Analysis

Transcriptomic analyses were conducted to explore the differentially expressed genes (DEGs) in males with/without winning experience. Acquisition of winning experience in the males followed the above mentioned protocol. Each treatment included three replicates, and each replicate included 15 male adults. At 11:00 a.m., the whole bodies of males were pooled in a plastic tube (1.5 ml) and snap-frozen in liquid nitrogen. Then, TRIzol Reagent (Invitrogen) was used to extract the RNA from each sample group, and 3 μg of total RNA from each sample was converted into cDNA using a NEBNext Ultra RNA Library Prep Kit for Illumina (NEB). Six cDNA libraries were constructed and subsequently sequenced with the Illumina HiSeq 2000 platform by Beijing Biomarker Technologies Co., Ltd., yielding raw reads. Approximately 7.5 Gb of paired-end reads was produced for each RNA-seq sample. Low-quality reads, poly-N reads and adapter sequences from the raw data were removed using a FASTX-Toolkit (http://hannonlab.cshl.edu/fastx_toolkit/). Raw sequence data generated were deposited into the Sequence Read Archive database of the NCBI with the accession no. PRJNA613859. Approximately 31.4 million clean reads were obtained from each sample, which contained more than 9.3 billion bases, and the mean GC content was 36.09% (34.98–37.09%) in each sample. The Q30 percentages were higher than 88.42% in each sample, which showed that the sequencing of each sample was of high quality. Then, the acquired clean reads were pooled and assembled using Trinity software (v2.5.1) (Grabherr et al. 2011). The longest transcripts belonging to each gene were chosen as the appropriate representatives to construct the unigene set. In total, 34,654 unigenes with lengths longer than 300 bp were generated, whereas 35.43% unigenes (12,278) were longer than 1 kb in length, and the N50 size was 2502 bp. Besides, the BUSCO tool was used to evaluate the transcriptome assembly (Simão et al. 2015). BUSCO completeness scoring of the assembly found 88.9% of the 1,658 core insecta genes [BUSCO summarized benchmark = C: 88.9% (S: 70.7%), F: 5.8%, M: 5.3%, *n*: 1,658).

For functional annotation, the pooled assembled unigenes were searched using BLASTX (v2.2.31) against public databases of Clusters of Orthologous Groups (COG), euKaryotic Orthologous Groups (KOG), Swiss-Prot, Clusters of Orthologous Groups (COG), egg NOG (v4.5), Protein family (Pfam), NCBI nonredundant protein sequences (nr), KEGG Ortholog database (KO), and Gene Ontology (GO), with an *E*-value cutoff of 10^–5^. In total, 25,038 unigenes were successfully annotated. Using our assembled transcriptome as a reference, we identified putative genes expressed in males with winning experience and males without any fighting experience by RSEM software using the fragments per kilobase of transcript per million mapped reads (FPKM) method (Li and Dewey 2011). DESeq2 package (v. 1.6.3) was used to differential expression analysis, which provided statistical routines for determining differential expression in digital gene expression data using a model based on the negative binomial distribution. Values of *P* were adjusted using the Benjamini and Hochberg’s approach for controlling the false discovery rate (FDR). Differentially expressed genes (DEGs) were selected based on the criteria of at least a twofold change (FC) and an FDR <0.01.

### Quantitative Real-Time Polymerase Chain Reaction

The results of the DEGs from transcriptomic analyses were evaluated and checked by quantitative real-time polymerase chain reaction (qRT-PCR). RNA from each sample group containing 15 males was extracted with TRIzol reagent (Invitrogen), and first-strand cDNA was synthesized using a PrimeScript RT Reagent Kit (TaKaRa). SYBR Green Real-Time PCR Master Mix (TaKaRa) was used for qRT-PCR analysis (ABI StepOne Plus; USA) following the manufacturer’s protocols. The 2^−ΔΔCt^ method was used to calculate the relative gene expression, and the translation elongation factor 1-α (EF1A) housekeeping gene was used as a reference. Primers for the selected DEGs and the EF1A gene are listed in Table 2 and were designed using Primer Express 2.0 software (http://www.idtdna.com/analyzer/Applications/OligoAnalyzer/).

**Table 2.** Primer pairs used for expression analysis using qRT-PCR

Gene name	Primer sequences
c63356.graph_c0	Forward: 5′- GAGCTTGCACAGATGCAGAA-3′
	Reverse: 5′-TGGAAGACCATTCCAGCACA-3′
c67551.graph_c0	Forward: 5′-CCTGTTGTTGATACCGCCTT-3′
	Reverse: 5′-TTGACGTGCAGAAGAAACCG-3′
c72181.graph_c1	Forward: 5′-AGTCTGCTCTTGGCCATCAT-3 ′
	Reverse: 5′-GACGATGCACGCTTTGAAAC-3′
EF1A	Forward: 5′-ACCACGAAGCTCTCCAAGAA-3′
	Reverse: 5′-AATCTGCAGCACCCTTAGGT-3′

### Effect of Dietary Supplementation on Winner Aggression

Based on the transcriptomic analyses, many genes related to energy metabolism were downregulated in the winner, which suggested that the amount of energy available was reduced after winning a contest. Thus, in this experiment, we tested the effect of dietary supplementation on winner aggression. As in the previous protocol, males acquired winning experience from 9:00 a.m. to 10:00 a.m. As most newly emerged males cannot immediately feed, both normal males without any fighting experience and winners were fed 30% honey water at 4 p.m. Then, individuals who fed were selected for subsequent aggression experiments lasting 3 h. Similar to the above protocol, four males were introduced into a cylindrical arena (height: 1 cm; diameter: 3.5 cm) containing a 1-d-old virgin female to estimate fighting intensity under the same conditions. The mean score of injuries per wasp and the proportions of injured males and severely injured males were calculated as the fighting intensity in each arena. In addition, at 4 p.m., the aggression of normal males and winners without dietary supplementation was also tested. Approximately eight to nine replicates for each treatment were performed.

### Statistical Analysis

All statistical analyses were performed using *R* software (version 3.1.1). The fighting intensity data were analyzed by the generalized linear model (GLM) using the lme4 package. Proportion data typically have nonnormally distributed error variance (Pickering et al. 2000, Briffa et al. 2013). Thus, when proportion data, including proportions of injured and severely injured males, in our study were analyzed, the model assumed a binomial error structure and used a logit link function. Where the data exhibited overdispersion (i.e., ratio of residual deviance and df > 1), significance testing was performed using the quasi-binomial error distribution to correct for the overdispersion (Crawley 1993, Wilson and Hardy 2002). We used the measure of fighting intensity as the response variable for each model, including male experience and/or dietary supplementation as a fixed effect. When testing interactions, the criterion for significance was P < 0.01 (Crawley 2007). Besides, DEGs were identified using the DESeq2 package (v. 1.6.3) (Anders and Huber 2010). The qRT-PCR data comparing gene expression in males without any fighting experience and with winning experience were analyzed with an independent *t*-test.

## Results

### Effect of Winning Experience on Aggression

**Fig. 1.**
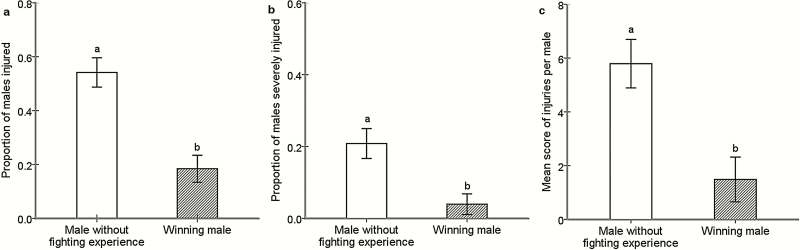
Effect of winning experience on aggression. Aggression in our studies was described with intensity of fighting. Fight intensity was assessed by the proportion of males (A), the proportion severely injured (B), and the mean score of injuries per wasp (C) after 3 h. The error bars indicate standard errors. The same letter on the column indicates no significant difference; however, different letters indicate significant difference.

For acquiring mating opportunities, males of *A. disparis* (i.e., without any fighting experience and without any dietary supplementation) engaged in extreme fighting behavior, resulting in 54.17 ± 5.44% males being injured and 20.83 ± 4.18% being severely injured. Fighting intensity of four males with winning experience in the arena was significantly lower than that of four males without any fighting experience, which was measured by the proportion of injured (Fig. 1A: Wald X12 = 13.778, P < 0.001) and severely injured males (Fig. 1B: Wald X12 = 9.174, P = 0.002) and mean score of injuries per wasp (Fig. 1C: Wald X12 = 13.058, P < 0.001).

### Transcriptomic Analysis

**Fig. 2.**
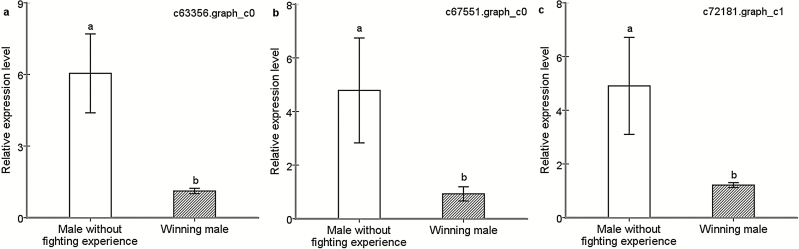
Expression of annotated genes involved in sugar transporter (A and B) and dopamine synthesis (C). The expression of genes determined through qRT-PCR was calculated by the 2^−ΔΔCt^ method using the housekeeping gene EF1A as a reference to eliminate sample-to-sample variations in the initial cDNA samples. The error bars indicate standard errors. The same letter on the column indicates no significant difference; however, different letters indicate significant difference.

Twenty-two unigenes were differentially expressed between normal males and winners. Respectively, 1 and 21 annotated genes were up- and downregulated in winners. Functional annotation showed that the upregulated gene (i.e., c69976.graph_c0), in winners mainly is associated with the lipid transport and metabolism. Many downregulated genes are related to energy metabolism, for example, energy production and conversion and sugar transport (e.g., c42247.graph_c1, c58186.graph_c0, and c17803.graph_c0; Supp Table 1 [online only]). Two annotated genes were selected to check the results from transcriptomic analyses by qRT-PCR (Fig. 2A and B), and the results showed consistency. Combined with the transcriptomic analyses, genes (c63356.graph_c0; c67551.graph_c0) related to sugar transporter were downregulated (Fig. 2A and B: evaluated by qRT-PCR, c63356.graph_c0, *t* = −2.98, df = 6, *P* = 0.012; c67551.graph_c0, *t*=-1.96, df = 6, *P* = 0.033).

### Effect of Dietary Supplementation on Winner Aggression

**Fig. 3.**
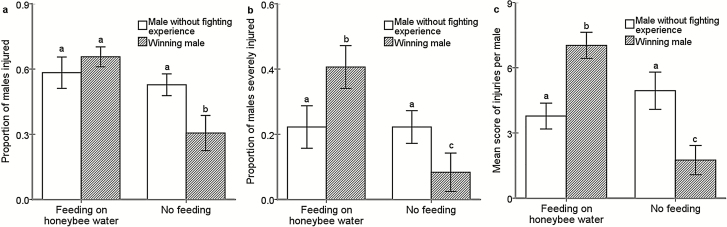
Effect of dietary supplementation on winner aggression. Aggression in our studies was described with intensity of fighting. Fight intensity was assessed by the proportion of males (A), the proportion severely injured (B), and the mean score of injuries per wasp (C) after 3 h. The error bars indicate SEs. The same letter on the column indicates no significant difference; however, different letters indicate significant difference.

Winners significantly increased their fighting intensity after dietary supplementation, resulting in 65.63 ± 5.63% injured and 40.63 ± 6.58% severely injured males (Fig. 3). The proportions of males injured (Fig. 3A: Wald X12=12.923, P < 0.001) and severely injured (Fig. 3B: Wald X12=7.203, P = 0.004) in winner combat after dietary supplementation was significantly higher than that in winner combat without any dietary supplementation, which was also measured by the mean score of injuries per wasp (Fig. 3C: Wald X12 = 32.128, P < 0.001).

In addition, except for the proportion of injured males (Fig. 3A: Wald X12 = 0.386, P = 0.537), the results showed that the fighting intensity of winners after dietary supplementation was higher than the fighting level of normal males, which was measured by the proportion of severely injured males (Fig. 3B: Wald X12 = 3.465, P = 0.035) and mean score of injuries per wasp (Fig. 3C: Wald X12 = 16.971, P < 0.001). Furthermore, there was an interaction effect between dietary supplementation and type of male on the proportion of severely injured males (Wald X12 = 7.88, P = 0.008) and mean score of injuries per wasp (Wald X12 = 19.787, P < 0.001) but not for the proportion of injured males (Wald X12 = 3.347, P = 0.074).

## Discussion

Prior fighting experience is one of the factors influencing fighting behavior in many species (Whitehouse 1997, Hsu and Wolf 1999, Jennings et al. 2004, Reaney et al. 2011, Schwartzer et al. 2013, Goubault and Decuigniere 2012, Goubault et al. 2019). Empirical reports have proposed that prior winning fighting experiences increase the willingness and frequency of aggressive acts (Whitehouse 1997, Khazraie and Campan 1999, Hsu and Wolf 2001, Hsu et al. 2006). However, our results showed that fighting intensity of four males with winning experience in the arena was significantly lower than that of four males without any fighting experience. It suggested that *A. disparis* males with winning fighting experience (before diet supplementation) seem to be unwilling to attack each other, resulting in a decrease in fighting intensity, which was inconsistent with many empirical reports. Transcriptomic analyses showed that many genes related to energy metabolism were downregulated in winners. It was suggested that although individuals won the contest, most of their energy might have been consumed in previous fights. Aggression has been considered an ‘energetic war of attrition’, where the winning contestant is the one that commits the greatest amount of energy to the contest (Payne and Pagel 1997, Briffa and Elwood 2004, Copeland et al. 2011, Briffa 2014). Rather than the ‘give up’ or ‘retreat’ phenomenon observed in many species that exhibit nondangerous fighting (reviewed by Enquist and Leimar 1990), male fighting in *A. disparis* follows a dangerous pattern, ending with contestants being severely injured or killed (Liu et al. 2017a, Liu and Hao 2019). Thus, in *A. disparis*, winning the contest would not only allow the winner to acquire mating opportunities but also to protect himself from injury. Consequently, the winner might consume great energy in order to win the contest.

Although individuals won the contest, our results showed that in *A. disparis*, winners decreased their fighting intensity for subsequent contests, which was inconsistent with many empirical reports (Reviewed by Hsu et al. 2006, Goubault and Decuigniere 2012, Goubault et al. 2019). Most energy consumption in previous fights was likely to constrain the winner’s intensity during subsequent fights and influence strategic decisions. Interestingly, after dietary supplementation, winners seemed to increase their fighting intensity in subsequent contests. One explanation was that winners with low-energy reserves might not have enough energy to attack others in subsequent contests, resulting in reduced fighting intensity (Snart et al. 2018). Alternatively, males of *A. disparis* frequently engage in dangerous fighting behavior (Liu et al. 2017a, Liu and Hao 2019); thus, the costs of fighting are usually substantial (Lane and Briffa 2017). Empirical evidence has shown that individuals are expected to monitor the costs and benefits associated with a contest and adjust their fighting behavior accordingly (Clutton-Brock et al. 1979, Dawkins and Brockmann 1980, Barnard and Brown 1984, Stockermans and Hardy 2013). As potential fighting costs are high for *A. disparis* and the possibility of a win for a previous winner with low-energy reserves is slim in a subsequent fight, winners might be unwilling to attack others to avoid revenge by opponents and thus show decreased fighting intensity to escape injuries.

In addition, our results further showed that the fighting intensity of winners after dietary supplementation was significantly higher than that of normal males without any fighting experience. Usually, fighting experience should be integrated with other factors to predict the final outcome of fighting, which, for example, is hypothesized to affect a contestant’s fighting behavior by altering its estimated fighting ability (Parker 1974, Beaugrand et al. 1991, Hsu and Wolf 1999, Mesterton-Gibbons 1999, Hsu et al. 2006). Specifically, for example, prior winning fighting experience may increase an individual’s actual fighting ability or perceived fighting ability (Beacham 1988, Hsu et al. 2006, Goubault and Decuigniere 2012, Goubault et al. 2019). In *A. disparis*, winning increased fighting skill and ability from previous contests, which might further increase the fighting intensity of the winner after dietary supplementation.

In this study, *A. disparis* was used as an experimental model to study the effect of winning fighting experience on aggression. The results suggested that although winners increased their fighting ability from previous contests, available energy was likely to be a crucial factor affecting the intensity and strategic decisions of subsequent fights. In addition, our transcriptomic analyses also showed that a decrease in neuroendocrine dopamine (DA) might be a physiological contributor to winner modification of subsequent fighting behavior. An annotated gene (Supp Table 1 [online only]: c72181.graph_c1) encoding tyrosine 3-monooxygenase (TH) was downregulated in winning males (Fig. 2C: evaluated by qRT-PCR, *t* = −2.04, df = 6, *P* = 0.038); this gene encodes what is thought to be the rate-limiting enzyme in dopamine synthesis (Budnik and White 1987). Similar to serotonin (5-hydroxytryptamine [5-HT]), DA is an important neurotransmitter and is thought to be related to aggression in many species (de Almeida et al. 2005, Alekseyenko et al. 2013). An increasing number of studies have shown that physiological changes in hormone, neurohormone, and biogenic amine systems modulate behavioral changes after winning and losing experiences (Hannes et al. 1984, Huhman et al. 1991, Schuett et al. 2000, Hsu et al. 2006, Schwartzer et al. 2013, Mathiron et al. 2019). However, whether dopamine has an effect on aggression in *A. disparis* and whether it is induced by experience to modify the winner’s fighting behavior are unclear and should be further studied.

## Supplementary Material

ieaa038_suppl_Supplementary_Table_S1
